# Imaging biomarkers in functional neurological disorders

**DOI:** 10.1098/rstb.2024.0472

**Published:** 2026-09-17

**Authors:** Irene Lozzi, Cristina Concetti, Selma Aybek Rusca

**Affiliations:** ^1^ Department of Neuroscience, Biomedicine and Movement, University of Verona, Verona, Italy; ^2^ Faculty of Sciences and Medicine, University of Fribourg, Fribourg, Switzerland

**Keywords:** functional neurological disorder, fMRI, structural MRI, imaging biomarkers, neurology

## Abstract

Recent advances in neuroimaging have significantly reshaped our understanding of functional neurological disorder (FND), shifting the focus from structural damage to dysfunctional brain networks. Building on Charcot's early intuition of a ‘functional lesion’, this review traces the evolution of neuroimaging research in FND, from its historical roots to current developments. We synthesize findings from structural and functional imaging studies, highlighting alterations in brain regions involved in motor control, self-agency and emotion regulation. Task-based fMRI studies have shown atypical activation in areas such as the supplementary motor area, dorsolateral prefrontal cortex and temporo-parietal junction, supporting the notion of impaired voluntary motor control. Additionally, changes in the amygdala and periaqueductal grey point to the role of emotional dysregulation. Structural imaging reveals grey and white matter alterations, while machine learning applied to resting-state connectivity shows promise for biomarker development. Emerging evidence also suggests that genetic and epigenetic factors—particularly within oxytocinergic and serotonergic systems—may contribute to individual vulnerability. We argue for a multi-modal approach integrating neuroimaging, genetics and computational methods to advance both the diagnosis and treatment of FND.

This article is part of the discussion meeting issue ‘Digital healthcare for the management of functional neurological disorders’.

## 1. Introduction

Functional neurological disorder (FND) is a condition characterized by distinct patterns of genuinely experienced symptoms and signs, which fluctuate over time both between the same task and across different tasks [1]. These symptoms, which can affect motor, sensory and cognitive functions, have long posed challenges in both diagnosis and treatment. Unlike classical neurological disorders, FND lacks a clearly identifiable structural lesion, leading to historical misconceptions and debates regarding its aetiology. Today, growing evidence supports the idea that FND arises from disruptions in brain function rather than irreversible damage, yet no definitive imaging biomarker has been established.

The modern understanding of FND has its roots in the late nineteenth century, particularly in the work of Jean-Martin Charcot. As a pioneering neurologist, Charcot was among the first to systematically study what was then termed ‘hysteria’ at the Salpêtrière Hospital in Paris. He sought to approach the condition with the same rigour as other neurological diseases, using clinical observation, hypnosis and early forms of neurophysiological investigation. In 1889, Charcot famously stated that in hysteria, ‘there is without doubt a lesion at some level of the nervous system, but certainly not of the nature of a circumscribed organic lesion of destructive nature’, emphasizing that this dysfunction eluded the anatomical methods available at the time. His notion of a lesion that was not structural in the classical sense but instead functional anticipated modern concepts of neural network dysfunction.

While Charcot's ideas were later overshadowed by psychoanalytic theories, the advent of neuroimaging has revived interest in understanding FND as a disorder of brain function. Over the past few decades, advances in magnetic resonance imaging (MRI)-based techniques have provided unprecedented insights into the neural mechanisms underlying FND. Although no single imaging biomarker has been identified, research has moved from descriptive clinical observations to sophisticated analyses of brain structure, task-based activation and resting-state connectivity. Additionally, emerging studies suggest that genetic and epigenetic factors may contribute to individual vulnerability to FND.

This article will trace the evolution of neuroimaging research in FND, from its historical foundations to the latest advancements, by taking inspiration from Charcot's shift in perspective—from asking where the lesion is, to exploring what kind of lesion it might be—as a guiding framework for our investigation [2]. By synthesizing findings across structural and functional imaging, as well as exploring biological predisposition, we aim to highlight the progress made in identifying biomarkers and improving our understanding of FND.

## 2. A dynamic lesion

The first evidence supporting the dynamic and reversible nature of cerebral blood flow alterations in FND emerged from single-case studies. One of the earliest studies using single-photon emission computed tomography identified a reduction in cerebral blood flow within the right parietal cortex during the presence of sensory-motor symptoms [3]. This initial observation laid the foundation for subsequent neuroimaging investigations aimed at elucidating the neural mechanisms underlying FND. A few years later, a study employing positron emission tomography provided additional insights, showing increased activation in the orbitofrontal cortex (OFC) and anterior cingulate cortex in a patient with FND when they were asked to move the affected limb [4]. Notably, there was a conspicuous absence of activation in the primary motor cortex (M1). The role of volition and agency in FND started to emerge from these findings, pointing to symptoms that result from disruptions in neural control mechanisms rather than deliberate action. Nevertheless, for many years, FND patients were erroneously regarded as intentionally producing their symptoms.

*Simulated or real?* Building on this perspective, neuroimaging studies have challenged historical misconceptions about symptom presentation in individuals with FND, often attributed to feigning or malingering and therefore implying intentional simulation [5]. Indeed, functional neuroimaging has consistently shown that FND exhibits distinct activation patterns that diverge from those observed in individuals simulating symptoms. Research comparing brain activity in FND patients and individuals instructed to feign symptoms has demonstrated key differences in neural engagement. While patients, irrespective of symptom lateralization, show decreased activation in the left dorsolateral prefrontal cortex, a region associated with deliberate control and volition, this pattern is notably absent in feigners [6]. Further evidence from functional magnetic resonance imaging (fMRI) studies in motor execution and inhibition tasks has corroborated the evidence that patients with functional weakness exhibit specific activation patterns that, while sharing some similarities with those observed in simulated weakness, remain fundamentally distinct. This pattern of brain activity suggests that patients engage in more complex mental processes than feigners, further supporting the notion that FND symptoms arise from unique neural mechanisms rather than conscious control [7–9].

## 3. Functional magnetic resonance imaging

### (a) Motor control and sense of agency in functional neurological disorder

A growing body of research has demonstrated alterations in brain regions involved in self-monitoring, motor preparation and the sense of agency in patients with FND [10–13]. One influential study examined brain responses during a motor imagery task in patients with functional paralysis, which were asked to imagine movements without actual execution while undergoing fMRI [11]. The findings revealed that patients, while engaged in imaginary actions with the affected hand, exhibited significantly increased activation in the ventromedial prefrontal cortex (vmPFC) compared to the activation observed for the unaffected hand. This region, a central node in the default mode network (DMN) and associated with self-referential processing and self-monitoring, may reflect a compensatory or maladaptive mechanism [14,15]. The heightened vmPFC activity is thought to be indicative of heightened internal monitoring processes [11].

Further studies examining motor preparation in FND patients revealed abnormalities in the supplementary motor area (SMA). A study employing both internally and externally generated movement paradigms (i.e. freely chosen or cue-driven) reported diminished activation in the SMA, as well as higher activation in limbic regions among patients [16]. Notably, the reduced SMA activity was present regardless of the movement initiation mode. Furthermore, the study demonstrated impaired functional connectivity between the SMA and the dorsolateral prefrontal cortex (DLPFC), specifically during internally generated actions. This disruption in connectivity suggests the presence of deficits in top-down control processes, where frontal executive regions fail to adequately regulate motor planning and execution. This happens selectively for internally generated actions, under attentional control, and less for externally generated actions, which are automatic. This seems to correspond to what is observed clinically in patients who exhibit ‘motor discordance’: being able to walk and put weight on a weak leg (automatic movement) but unable to lift it from the examination bed (voluntary movement requiring attention) [1].

Sensorimotor integration could also be affected by abnormal interoceptive processes in FND. In a recent task-based fMRI study, researchers investigated cardiac interoceptive processing in patients with FND. During a heartbeat-counting task, patients demonstrated decreased activation in the right anterior insula, dorsal anterior cingulate cortex and temporoparietal junction (TPJ) when attention was directed to interoceptive signals [17]. Importantly, reduced activation in these salience network regions was associated with lower interoceptive accuracy and altered interoceptive trait prediction error (ITPE), an index of discrepancy between subjective belief in one's interoceptive ability and objective interoceptive accuracy [18,19]. Decreased vmPFC activation in association with heightened ITPE may reflect a diminished capacity to assign appropriate meaning to internal bodily signals—a mechanism that could underlie both interoceptive and agency disruptions [17]. This convergence is particularly relevant, given the notion that accurate internal state monitoring is critical for updating motor commands and establishing a coherent sense of agency [20,21].

Indeed, several studies have provided compelling evidence for alteration of the sense of agency in FND. In a fascinating study, agency perception was manipulated using a cyber-glove paradigm to artificially alter the perceived control over hand movements so that patients experienced a mismatch between their intended movements and the visual feedback received [22]. Unlike healthy controls, individuals with FND exhibited a lack of modulation in right TPJ activity, suggesting a failure in integrating sensory and motor information necessary for agency attribution. Similar findings were reported by Baek *et al.* [10] using a Libet's clock task, further reinforcing the hypothesis that agency attribution is impaired in FND.

Not only task-based, but also resting-state functional connectivity studies have provided additional insights into TPJ dysfunction in FND. Researchers identified decreased connectivity between the right TPJ and somatomotor regions, indicating a possible disconnection between sensorimotor integration and higher-order cognitive processing, thereby impairing the sense of agency [23]. By contrast, a more recent study applying connectivity-centrality analyses found increased centrality values for the left TPJ in patients with functional weakness [24]. The higher centrality may reflect a compensatory reorganization or an imbalance in lateralized TPJ contributions, further complicating the neural picture of agency deficits in FND.

Beyond observational studies, neuromodulatory approaches have begun to test causal hypotheses regarding these neural circuits. In a study using excitatory transcranial magnetic stimulation (TMS) to target the right TPJ, researchers observed that hypoactivation in this region during an agency task could be transiently restored [25]. However, the increased TPJ activation induced by TMS did not translate into immediate clinical improvement; it is also possible that such a stimulation protocol may not suffice to induce measurable behavioural changes and that repeated or more prolonged interventions might be necessary. Rather than refuting a role for the TPJ in agency, these findings suggest that the TPJ activation alone may be necessary but not sufficient to restore normal agency processing. Abnormalities in the preparatory and monitoring phases of voluntary action engage prefrontal and premotor regions in addition to the TPJ, suggesting that disruptions in agency may stem from altered top-down processes, as well as faulty sensory integration [10]. Likewise, the decreased TPJ–somatomotor connectivity reported by Maurer *et al.* [23] points to a breakdown in the reciprocal exchange of information between higher-order and sensorimotor systems. The absence of behavioural improvement despite neural normalization likely indicates that the sense of agency cannot be ascribed to the TPJ in isolation, but emerges from and depends on the integrity and coordination of a broader network.

In conclusion, early neuroimaging findings provided critical groundwork for conceptualizing FND as a disorder characterized by network dysregulation rather than a focal neurological deficit. Modern imaging approaches have expanded on these early insights, shifting the focus from simple anatomical localization to the dynamic interplay between functional circuits involved in motor control and emotion regulation. Disruptions in top-down motor control mechanisms, involving regions such as the vmPFC, DLPFC and SMA, appear central to the pathophysiology of FND. At the same time, impairments in agency perception, linked to dysfunction in the TPJ and insula, likely further contribute to the motor symptoms experienced by patients.

### (b) Emotion processing in functional neurological disorder

While much of the early neuroimaging research in FND focused on motor control, recent studies have shifted attention towards the neural correlates of emotion processing. On the one hand, this shift aligns with long-standing theories positing that psychogenic factors play a central role in the manifestation of FND symptoms, while on the other hand, it extends beyond these frameworks by highlighting additional processes and complexities. One of the first imaging studies in this domain investigated amygdala responses to emotional facial expressions [26]. Whereas healthy participants displayed the expected pattern of greater right amygdala activation to fearful than to happy faces, FND patients failed to show a valence-dependent differentiation. Specifically, compared with controls, they exhibited heightened right amygdala activation to happy faces but no significant differences when viewing fearful faces. Time-course analyses further revealed reduced habituation of amygdala responses to emotional stimuli, whereas effective connectivity analyses demonstrated an increased directional influence from the right amygdala to the SMA. According to the authors, the failure of limbic habituation is likely to result in a persistently elevated amygdala output, that can, in turn, aberrantly engage motor preparatory regions. Heightened affective signals, whether positive or negative, may thus trigger motor system activity in FND through increased limbic-motor coupling, rather than a simple amplification of emotional reactivity.

A further study of emotional processing in FND incorporated sad facial expressions into the original paradigm [27]. Results demonstrated an increased activation of the amygdala, that did not show habituation over time like in healthy controls but even a tendency to further increase, showing again a state of hyperarousal. In addition, this study showed increased activity in patients in the periaqueductal grey (PAG), a brainstem region known for its involvement in interoceptive processes, particularly in regulating breathing in the freeze response [27]. This warrants further studies into the mechanisms of automatic defence behaviour. Additional evidence implicating the amygdala in FND-related emotion processing was provided by a study investigating cognitive reappraisal mechanisms with fMRI [28]. Results revealed heightened right amygdala activation in FND patients during negative cognitive reappraisal, further underscoring the role of limbic hyperactivity in the emotional responses of these individuals.

Complementary evidence for emotion regulation deficits in FND emerges from studies examining insular function. Patients with FND showed reduced activation of the insula during an emotion regulation task [29]. Interestingly, there was an inverse correlation between insular activity and alexithymia scores, suggesting that individuals with greater difficulty in identifying and describing emotions exhibited the most pronounced activity deficits. These findings provide a potential mechanistic link between altered interoception and emotional dysregulation in FND.

Beyond motor dysfunction, research has increasingly highlighted the role of limbic structures, including the amygdala, PAG and insula, in mediating aberrant emotional processing in FND. These findings suggest that emotional dysregulation and altered interoceptive awareness may be integral components of the disorder.

### (c) Static and dynamic functional connectivity studies

Moving from ‘where is this dynamic lesion’ to ‘what is the nature of this lesion’, the field of neuroimaging moved from tasks and localization to network analysis. Several resting-state fMRI (rs-fMRI) investigations have employed static functional connectivity approaches, examining how strongly different brain regions are functionally linked on average over time by analysing correlations in their blood-oxygen-level-dependent (BOLD) signals [30,31]. Differences were also noted in the DMN, with some findings reporting decreased connectivity between key DMN hubs (e.g. posterior cingulate cortex, medial prefrontal cortex) and subcortical areas involved in motor and affective processing; additionally, salience network connectivity appeared altered in several studies, especially involving regions such as the insula and dorsal anterior cingulate cortex, potentially indicative of abnormal integration among emotional, attentional and motor processes [32,33]. Alternative, dynamic approaches to functional connectivity have further refined our understanding by assessing temporal fluctuations in BOLD signal synchronization and capturing how patterns of connectivity between brain regions change over time, rather than assuming they remain static throughout the scan. Among these, a novel approach involves identifying co-activation patterns (CAPs) that recur over short time intervals [34]. In FND samples, a set of significant CAPs has been reported to differ in both frequency and duration when compared with healthy controls [35]. Notably, some of these altered patterns reflect heightened co-activation among regions implicated in salience detection (e.g. anterior insula and dorsal anterior cingulate cortex) and sensorimotor function (e.g. primary motor and sensory cortices), possibly pointing to an atypical tendency towards either initiating or inhibiting motor output. In addition, certain CAPs alterations were jointly associated with amylase levels and symptom duration in patients, suggesting that they may linger in specific connectivity states, potentially influencing emotional reactivity or contributing to inconsistent motor control [35]. Such findings have been interpreted as indicative of reduced network flexibility that may be relevant to the intermittent or episodic clinical features observed in FND.

Although the precise clinical implications of these network-level changes are still under exploration, these insights illustrate a paradigm shift in neuroimaging research on FND, from attempting to isolate a focal site of dysfunction to examining how entire networks and their dynamic fluctuations may contribute to symptom emergence and maintenance.

## 4. Structural neuroimaging studies

After considering the ‘dynamic’ and non-localized nature of lesions in FND, seeking structural differences between individuals with FND and healthy controls may appear contradictory. Nonetheless, structural neuroimaging studies have reported significant alterations in both grey and white matter, suggesting that these changes could influence symptom development.

Voxel-based morphometry analyses have identified significant differences in grey matter volume (GMV) among individuals with FND. One study reported increases in GMV within the left amygdala, left caudate, left putamen, left cerebellum, left fusiform gyrus and bilateral thalami, alongside decreases in the left sensorimotor cortex [23]. These areas play key roles in emotional processing and motor function, implying that structural differences in these regions could be relevant to FND symptoms. Of note, these volumetric changes did not correlate with anxiety or depression scores, suggesting specificity to FND rather than comorbid psychiatric conditions. In paediatric populations, another investigation found larger GMV in the left SMA, right superior temporal gyrus and dorsomedial prefrontal cortex (DMPFC) among children and adolescents with FND [36]. The SMA, especially its pre-SMA region, is implicated in motor planning and the integration of cognitive and emotional inputs, indicating that structural alterations in this area may impact motor function in FND. Furthermore, increased volumes in the SMA and DMPFC were associated with faster emotion identification, pointing to a possible relationship between structural differences and emotional processing in younger patients.

Concerning white matter alterations, diffusion tensor imaging studies have revealed microstructural differences in white matter tracts among FND patients. Despite an early study which found increased fractional anisotropy (FA) in the left uncinate fasciculus among patients with psychogenic non-epileptic seizures [37], recent evidence points to more widespread and predominantly reduced FA across multiple limbic-prefrontal pathways. Indeed, researchers found reduced FA in pathways including the stria terminalis/fornix, medial forebrain bundle, extreme capsule, uncinate fasciculus, cingulum bundle, corpus callosum and striatal-postcentral gyrus projections [12]. These tracts are associated with salience processing, defensive behaviours and emotion regulation [38]. The study also reported that decreased FA in the stria terminalis/fornix and medial forebrain bundle correlated with greater physical disability and longer illness duration, suggesting that white matter integrity may reflect disease severity and progression in FND [12]. In a subsequent study using probabilistic tractography, the authors further confirmed widespread FA reductions, including in the uncinate fasciculus, reinforcing its role in limbic-prefrontal communication and its potential vulnerability in FND [32].

Putting these points into perspective, these findings highlight that, even within a ‘dynamic’ framework, observable neuroanatomical variations do exist in individuals with FND. However, it remains crucial to distinguish between trait and state markers when interpreting these findings. Trait markers may reflect inherent vulnerabilities or predispositions that exist prior to the development of FND, whereas state markers refer to neuroanatomical changes that emerge in response to symptom presence and can fluctuate accordingly. For instance, some studies reported increased GMV in the amygdala and premotor cortex [36,39,40]. While the former focused on children with FND, the latter two examined adult samples, indicating that enlarged volumes are not limited to early developmental stages. Although these did not explicitly propose these findings as a trait marker, one plausible interpretation could be that such structural alterations reflect regionally specific compensatory or adaptive mechanisms supporting emotion recognition, regulation or sensorimotor control. Conversely, a recent study in adults reported reduced bilateral amygdala volume as a trait marker in patients with FND [41]. This variability may possibly stem from the developmental sensitivity of the amygdala to early life stressors [42,43], highly prevalent among individuals with FND [44] which could interact with later stress exposure to shape divergent volumetric outcomes [45–48].

Although GMV does not show a uniform direction of change across studies, its deviation from normative patterns may nonetheless represent a trait-like index of altered neurodevelopmental plasticity. Rather than the sign of the difference, it is the presence of atypical adaptation trajectories that may confer vulnerability to FND. On the other hand, white matter alterations, particularly reduced FA in limbic-prefrontal tracts, such as the uncinate fasciculus and the stria terminalis, may function as state markers in FND. Their association with illness duration and functional impairment points to dynamic, experience-dependent neuroplastic changes, evolving with symptom progression and potentially responding to therapeutic interventions [49]. Conceptually, these neurobiological dimensions could be arrayed along a continuum, from enduring anatomical predispositions to rapidly modifiable functional markers, capturing distinct levels of neural organization and flexibility shaping FND pathophysiology.

## 5. Diagnostic biomarkers: resting-state classifiers

While current clinical signs specific to FND are already highly reliable [50–52], it is conceivable that in the future, integrating biomarkers may further enhance diagnostic precision. The application of machine learning techniques to rs-fMRI data has emerged as a promising technique for identifying objective neurobiological markers that can complement or even surpass traditional diagnostic methods [53]. Classifiers trained on large datasets can potentially learn individual ‘fingerprints’ of dysfunctional connectivity, offering insights into symptom-specific network signatures and advancing diagnostic precision [54,55].

Indeed, investigations into rs-fMRI classifiers for FND demonstrated fair diagnostic success. In a first, pioneer investigation on a modest sample of patients and matched healthy subjects, whole-brain rs-fMRI data were acquired and used to conduct atlas-based functional connectivity analyses [56]. A support vector machine (SVM) classifier was then applied to the dataset, achieving an accuracy exceeding 68% (*p* = 0.004) in differentiating FND patients from controls. Notably, the most discriminative connectivity patterns involved regions such as the right caudate, amygdala, prefrontal and sensorimotor areas. Post hoc analyses further revealed hyperconnectivity in these regions among patients, suggesting that even early-stage applications of machine learning to rs-fMRI data can uncover meaningful neural alterations in FND [56]. Although this preliminary study was constrained by a small sample size and site-specific variations, it provided an important proof of concept that functional connectivity could serve as an objective marker of underlying pathophysiological processes.

Building on these initial findings, a subsequent multi-centre study expanded the sample size and diversity by collecting rs-fMRI data from FND patients and controls across four different centres in three different countries (Switzerland, The Netherlands and Czech Republic) [57]. This study employed a multi-variate machine learning framework based on whole-brain functional connectivity. Although individual centres yielded accuracies in the 70–73% range during replication steps, pooled cross-validation across centres produced an overall accuracy of 71.5% in distinguishing patients from healthy controls. The most discriminative features in this multi-centre analysis were found in regions including the angular and supramarginal gyri, sensorimotor cortex, cingulate cortex, insular cortex and hippocampal regions. However, challenges remained, as inter-centre validation sometimes resulted in accuracy levels that did not exceed chance, highlighting the difficulties in generalizing findings across different scanning protocols and populations [57].

Another study replicated these findings, focusing specifically on functional movement disorders (FMD) and employing a targeted machine learning strategy [58]. Sixty-six seed regions, selected based on previous literature, were used to compute a full correlation matrix. Recursive feature elimination then identified the most predictive connections, which were used to build an SVM classifier that achieved an impressive 80% accuracy. The most informative features underscored the importance of functional interactions between brain regions implicated in emotion, reward processing and sensorimotor integration, further supporting the notion that specific alterations in functional connectivity may be a hallmark of the disorder.

A further study has also integrated structural MRI data to complement functional connectivity measures, underscoring the interplay between brain structure and function in FND. Patients with mixed FND were differentiated from healthy controls with an accuracy of 66% (*p* = 0.005) and from psychiatric controls with an accuracy of 60% (*p* = 0.038) [59]. Key regions contributing to the statistically significant classifications included the cingulate gyrus, hippocampal subfields and amygdala. When focusing on the FMD subtype specifically, a classifier robustly differentiated these patients from healthy controls, achieving an accuracy of 72% (*p* = 0.002). However, the classifier could not reliably distinguish patients with FMD from psychiatric controls, and the functional seizures subtype could not be effectively classified against either control group.

Adding another layer to these multi-modal approaches, a recent study has also examined voxel-wise BOLD signal variability [60]. First, voxel-wise BOLD signal variability was computed for each participant and group differences were evaluated. FND patients exhibited higher variability in key networks, including the somatomotor, salience, limbic and dorsal attention networks, compared with controls. Furthermore, in a longitudinal follow-up of 47 patients over eight months, increased BOLD signal variability in the SMA was associated with clinical improvement, suggesting that SMA variability may serve as a state biomarker. Conversely, higher baseline variability in the left insula predicted a poorer clinical outcome. These findings emphasize the dynamic nature of neural activity in FND and support the potential of BOLD signal variability as both a prognostic and state marker, underscoring the intricate interplay between brain structure and function in the disorder. In line with this, an integrative diagnostic algorithm combining behavioural, neurophysiological and MRI potential biomarkers across different domains (e.g. motor, exteroceptive and interoceptive domains) has been proposed [61], with the aim of enhancing diagnostic precision and prognostic predictions.

Despite these promising advances, robust validation remains a major hurdle before clinical implementation. The heterogeneity of FND presentations, variations in scanning protocols and potential confounding comorbidities underscore the need for standardized methods. Going forward, large-scale consortia and open-data initiatives could further enhance the reliability of resting-state classifiers by harmonizing imaging parameters and promoting replication across diverse clinical populations.

## 6. Neurobiological, genetic and epigenetic contributions

The genetic underpinnings of FND remain largely unexplored, with only a limited number of studies investigating heritability and specific gene variants associated with the disorder. While no single gene has been definitively linked to FND, certain polymorphisms in genes related to neurotransmission, stress response and neurodevelopment have been observed in relation to the condition.

For instance, the G-allele of the rs1800629 polymorphism in the tumour necrosis factor-alpha (TNF-α) gene appears to be more frequent in healthy controls compared to individuals with somatoform disorders, suggesting a possible protective role of this variant [62]. By contrast, polymorphisms in genes encoding enzymes involved in monoaminergic metabolism, such as tryptophan hydroxylase and the catechol-O-methyltransferase (COMT) Val158Met variant, have not been found to differ significantly between patients with conversion disorder and healthy individuals [63,64].

Building upon the findings of a pioneer study demonstrating increased methylation of the oxytocin receptor gene (OXTR) in patients with FND [65], a recent work has also examined the rs53576 polymorphism of the same gene, a variant associated with social cognition and stress regulation [66,67]. Methylation changes in the OXTR gene have been reported in patients with FND, with higher methylation levels correlating with increased salivary oxytocin levels [68]. This finding suggests a possible compensatory mechanism, where reduced OXTR expression leads to increased oxytocin secretion as an attempt to maintain homeostasis. Moreover, given that oxytocin signalling is closely linked to limbic structures involved in emotional regulation, such as the amygdala and hippocampus [69,70], and that neuroimaging studies indicate that FND patients exhibit altered amygdalar connectivity patterns correlating with oxytocin levels, these findings strongly support the involvement of the oxytocinergic system in the disorder, potentially influencing emotional dysregulation frequently observed in the disorder ([68], p. 202).

Gene-environment interactions are increasingly recognized as crucial in the development of neuropsychiatric disorders [71,72], including FND. Epigenetic modifications, such as DNA methylation and histone acetylation, can induce long-lasting changes in gene expression without altering the DNA sequence [73–75] thereby mediating the effects of early-life adversity on brain function [76–78]. One of the most extensively studied epigenetic mechanisms in stress-related disorders involves DNA methylation of the glucocorticoid receptor (NR3C1) gene, which has been associated with a history of childhood trauma and reduced hippocampal volume [78]. Similarly, methylation of the serotonin transporter gene (SLC6A4) has been linked to lower serotonergic function in the OFC, potentially contributing to dysregulated emotional and interoceptive processing in FND [72].

The serotonergic system has also emerged as a critical factor in FND pathophysiology. Genetic variations in serotonin-related genes appear to influence symptom severity and clinical outcomes. For example, genetic polymorphisms in the tryptophan hydroxylase 2 (TPH2) gene, a crucial enzyme involved in serotonin synthesis, have been associated with both symptom severity and distinct neurobiological phenotypes in FMD patients. Specifically, carriers of certain TPH2 variants have demonstrated altered functional connectivity patterns within limbic-frontal circuits, correlating with heightened clinical severity and earlier disease onset [79]. Furthermore, a recent study identified a significant association between the rs1800532 polymorphism in tryptophan hydroxylase 1 (TPH1) and symptom severity, where carriers of the T allele exhibited more severe symptoms [80]. This polymorphism was linked to poorer clinical outcomes over an eight-month follow-up period, indicating a potential role of serotonin metabolism in illness progression. Additionally, a gene–gene interaction was identified between TPH2 (rs4570625) and OXTR (rs2254298), suggesting that the interplay between the serotonergic and oxytocinergic systems might contribute to symptom severity in FND patients [80]. These findings align with prior research implicating serotonin dysregulation in stress-related disorders and highlight its potential relevance in FND.

While research into the genetic and epigenetic contributions to FND remains in its early stages, existing findings highlight the importance of a multi-factorial model incorporating genetic predisposition, environmental exposures and neurobiological alterations. Future studies employing larger cohorts and integrative multi-omic approaches will be essential to further elucidate these complex relationships.

## 7. Conclusions and future directions

Recent findings from structural and functional imaging studies underscore abnormalities in motor control circuits, agency perception and emotional regulation, thereby reinforcing the hypothesis that FND reflects aberrant neural integration rather than overt cerebral injury. Concurrent genetic and epigenetic studies indicate that intrinsic biological factors may modulate vulnerability to FND, highlighting a multi-faceted interaction among molecular mechanisms, cognitive–affective processes and environmental influences.

Notwithstanding these advances, considerable challenges remain. One of the primary obstacles is the heterogeneity of FND presentations, which hinders the identification of reliable and generalizable biomarkers. Methodological heterogeneity in imaging techniques, sample sizes and acquisition protocols further compromises reproducibility across studies.

Future investigations would benefit from large-scale, multi-centre designs employing standardized imaging protocols to bolster both reproducibility and translational potential. Moreover, the integration of multi-modal approaches—encompassing neuroimaging, genetics, computational modelling and behavioural assessment—may facilitate a more comprehensive understanding of FND pathophysiology.

Beyond improving diagnostic precision, the exploration of neuromodulatory therapies constitutes a promising research trajectory. Preliminary applications of TMS and neurofeedback have shown potential to modulate aberrant neural circuitry; nevertheless, conclusive evidence for their therapeutic efficacy in FND is still lacking. Personalized treatment approaches tailored to individual neurobiological profiles, including functional connectivity-based biomarkers, may enable more targeted and effective interventions for specific patient subtypes.

Greater efforts are required to bridge the gap between neuroscience and clinical practice. While current diagnostic criteria for FND are predominantly based on clinical signs, incorporating objective neurobiological markers could enhance diagnostic confidence and improve patient management.

In conclusion, significant strides have been achieved in delineating the neural substrates of FND; however, additional research is imperative to translate these mechanistic insights into practical clinical applications. Ongoing efforts to refine diagnostic methodologies, innovate targeted treatments and foster interdisciplinary collaboration will be essential for advancing the FND research agenda.

## Data Availability

This article has no additional data.

## Theme

One contribution of 12 to a discussion meeting issue ‘Digital healthcare for the management of functional neurological disorders’.
